# Synthesis of modified 1,5-imino-d-xylitols as ligands for lysosomal *β*-glucocerebrosidase

**DOI:** 10.1007/s00706-019-02427-1

**Published:** 2019-05-11

**Authors:** Manuel Zoidl, Andreas Wolfsgruber, Michael Schalli, Seyed A. Nasseri, Patrick Weber, Arnold E. Stütz, Stephen G. Withers, Tanja M. Wrodnigg

**Affiliations:** 1 Institute of Organic Chemistry, Graz University of Technology, Graz, Austria; 2 Department of Chemistry, University of British Columbia, 2036 Main Mall, Vancouver, BC V6T 1Z1 Canada

**Keywords:** Carbohydrates, Conformation, Enzymes, *β*-Glucosidase ligands, Iminoxylitol, *β*-Glucocerebrosidase

## Abstract

**Abstract:**

Modified 1,5-dideoxy-1,5-imino-d-xylitol analogues with different substitution patterns involving position C-1 and/or the ring nitrogen were prepared, which were designed to serve as precursors for the preparation of iminoxylitol-based ligands and tools for the elucidation and modulation of human lysosomal *β*-glucocerebrosidase. Biological evaluation of the synthesized glycomimetics with a series of glycoside hydrolases revealed that these substitution patterns elicit excellent *β*-glucosidase selectivities.

**Graphical abstract:**

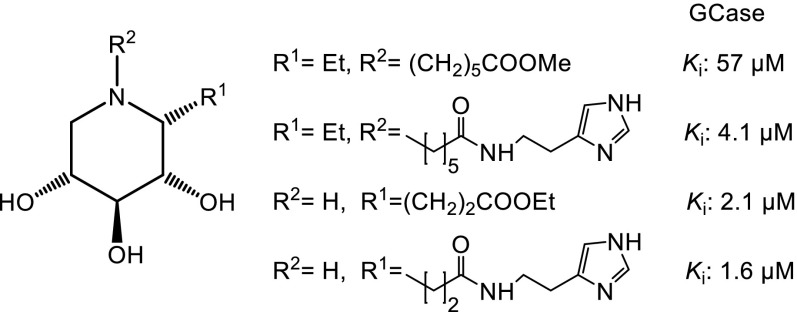

**Electronic supplementary material:**

The online version of this article (10.1007/s00706-019-02427-1) contains supplementary material, which is available to authorized users.

## Introduction

**Fig. 1:**
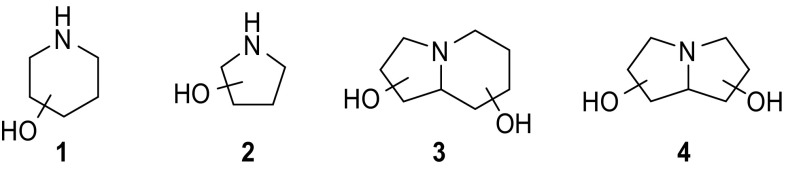
Structures of iminoalditol scaffolds **1–4**

Iminoalditols, also termed iminosugars, are natural occurring glycomimetics, in which the ring oxygen of the carbohydrate moiety is replaced by a trivalent basic nitrogen. Paradigmatic structural scaffolds are polyhydroxylated piperidines **1**, pyrrolidines **2**, indolizidines **3**, and pyrrolizidines **4** (Fig. 1) [1–5]. The nitrogen in the endocyclic position is responsible for the unique biological behavior of this compound class to interact and modulate active site specifically glycoside-processing enzymes. Since the last decades, such compounds have been of great interest for an interdisciplinary scientific community, including chemists, biochemists, as well as physicians.

Many different naturally occurring structures are known, exceeded by the number of synthetic derivatives, with manifold different modification patterns concerning the carbohydrate scaffold as well as customized derivatisations for different applications. This substance class has been implicated as potential therapeutic agents [6], for example, as immunomodulators [7, 8], as antibacterial [9, 10], antiviral [11, 12], anti-cancer [13], and anti-fungal [14] agents. In addition, iminoalditol-based glycomimetics have been identified as plant growth inhibitors [15]. An interesting field of application has emerged when iminoalditols have been applied at sub-inhibitory concentrations to act as protein-folding templates [16, 17] for mutant lysosomal enzymes, thus becoming candidates for the management of lysosomal storage disorders in the pharmacological chaperone therapy [18]. Moreover, this compound class has received great attention as probes for activity-based profiling of glycoside-processing enzymes [19–21].

**Fig. 2:**
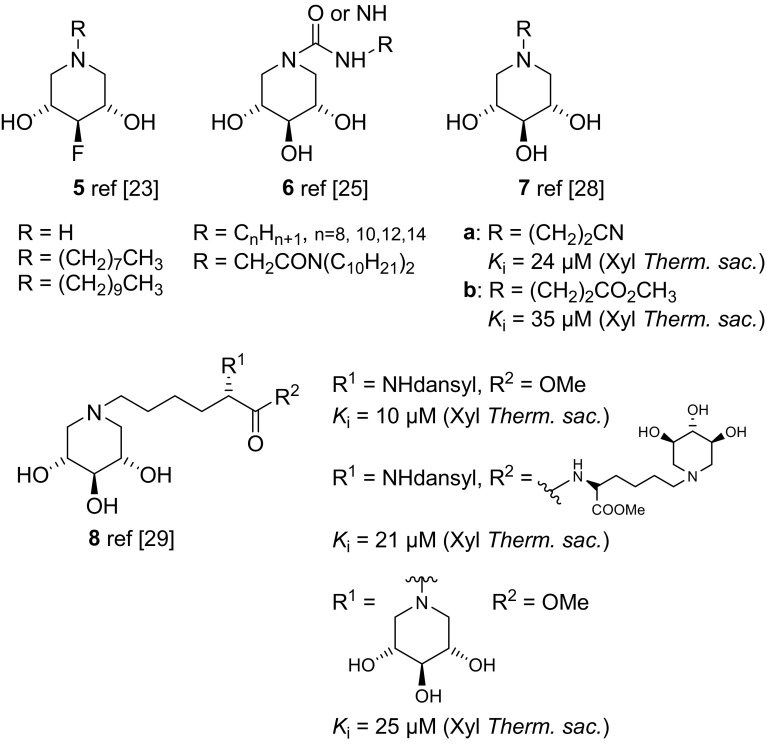
Structures of *N*-modified DIX derivatives **5–8**

The d-*xylo* configuration in the dideoxy iminoalditol scaffold has been shown to have very interesting ligand properties for glycoside-processing enzymes in terms of activity as well as selectivity [22]. Various modifications with respect to substituents as well as positions on the iminoxylitol scaffold have been synthesized and biologically investigated. Basically all of these compounds have been shown to be highly selective ligands for *β*-glucosidases. For example, fluorinated iminoxylitols carrying an *N*-alkyl group [23] (Fig. 2), such as compound **5**, have been found to exhibit immunosuppressive as well as glycosidase inhibitory activities. Based on Lehmann’s early finding [24], iminoxylitols bearing a guanidino or urea function at the ring nitrogen [25, 26], for example compound **6**, were synthesized and found to be selective inhibitors of human lysosomal *β*-glucocerebrosidase (GCase) with IC_50_ values in the low nm range. A deficiency of this enzyme causes Gaucher disease [27]. We have synthesized iminoxylitols modified at the endocyclic ring nitrogen with functionalized alkyl groups, such as compound structure **7** [28], as well as featuring more sophisticated substituents, including structure **8** [29]. These compounds exhibited inhibitory properties against *β*-xylosidase from *Thermoanaerobacterium saccharolyticum* (Xyl *Therm. sac.*), with *K*_i_ values in the lower µm range (Table 1).

**Fig. 3:**
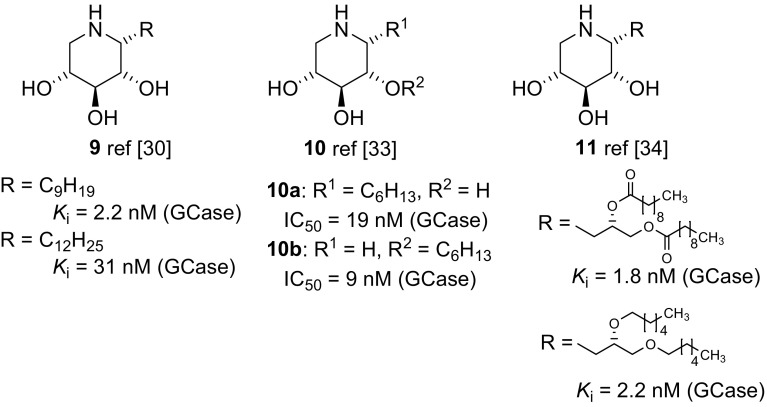
Structures of C-1 modified DIX derivatives **9–11**

Martin and co-workers have developed elegant synthetic routes towards 1-*C*-alkyl imino-d-xylitols **9** (Fig. 3), and showed that the introduction of the substituent at position C-1 improved the ligand properties as well as the selectivity for GCase significantly [30–32]. In addition, in a structure–activity study, the influence of the position of an alkyl chain has been investigated, showing that a 1–2 shift of the alkyl substituent from C-1 to O-2 (compounds **10a**–**10b**) increased the inhibitory property of the respective compound against GCase by a factor of 2 [33]. The same group has also synthesized 1,5-dideoxy-1,5-imino-d-xylitol (DIX) derivatives with alkyl substituents similar to ceramide at position C-1, for example compound **11**, and obtained highly potent GCase inhibitors which also showed selective chaperone properties for mutations associated to Types 1 and 2 Gaucher Disease [34].

**Fig. 4:**
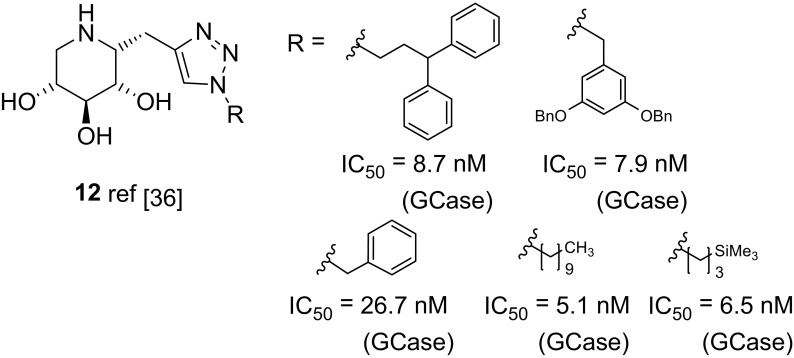
Structure of C-1 modified DIX derivatives **12**

Compain and co-workers synthesized a library of 1-*C*-triazolylalkyl side chain-modified DIX analogues (Fig. 4), including compounds **12**, by a click chemistry approach, and found that some of these are GCase enhancers for selected Gaucher disease genotype mutants [35, 36].

**Fig. 5:**
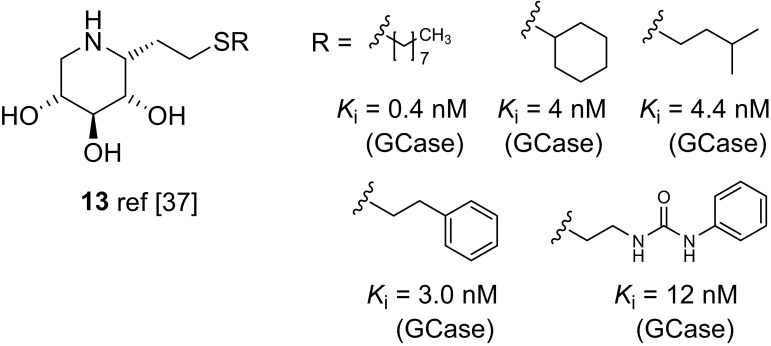
Structure of C-1 modified DIX derivatives **13**

Withers and co-workers developed a thiol-ene reaction sequence for rapid assembly of 1-*C*-alkyl DIX derivatives containing a sulfur atom between the DIX scaffold and the lipophilic substituent (Fig. 5), such as compounds **13**, and also found excellent ligand properties in terms of activity as well as selectivity for GCase furnishing promising potent and selective pharmacological chaperones for GCase mutants [37].

**Fig. 6:**
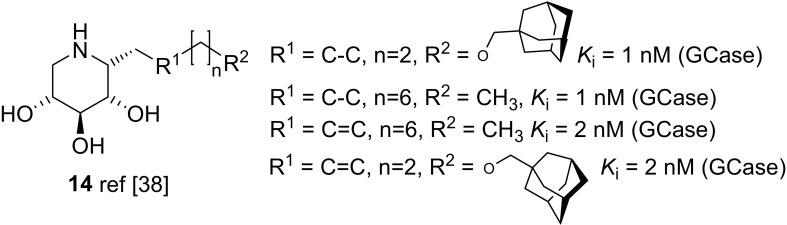
Structure of C-1 modified DIX derivatives **14**

Overkleeft and co-workers included into their structure–activity relationship study of lipophilic glycomimetics various d-*xylo* configured 1-*C*-iminosugar glycosides, for example compounds **14** (Fig. 6), and could demonstrate that these glycomimetics significantly exceed in terms of inhibitory activity as well as selectivity for GCase compared to the corresponding of d-*gluco* as well as l-*ido* configured analogues [38].

**Fig. 7:**
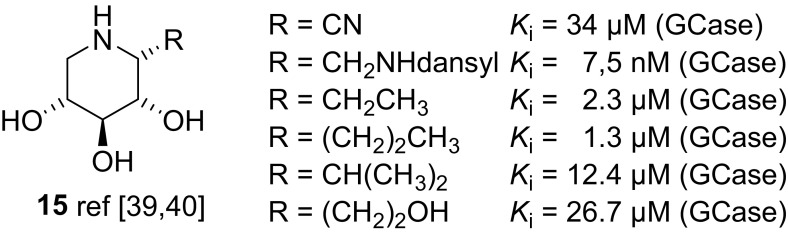
Structure of C-1 modified DIX derivatives **15**

We have developed a convenient synthetic protocol for the modification of the DIX scaffold at position C-1 taking advantage of the Staudinger/aza-Wittig/nucleophile reaction sequence [39, 40]. By this method, we have synthesized a range of simple C-1 alkyl modified DIX analogues **15** (Fig. 7) and have found the same trend for these compounds, which are highly selective ligands for GCase.

**Fig. 8:**
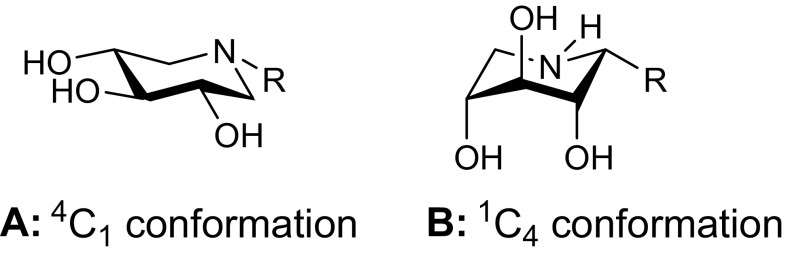
Conformations of C-1 modified DIX derivatives

All DIX derivatives carrying a substituent at position C-1, **9–15**, have been found to be locked in the ^1^C_4_ conformation when the alkyl substituent is introduced from the *β*-face at the pseudoanomeric center (**B**, Fig. 8). The hydroxyl groups at positions O-2, O-3, and O-4 are in an axial orientation and the substituent at position C-1 is equatorial due to a piperidine ring inversion under acidic conditions such as in the lysosomal environment. In contrast, ring nitrogen substituted DIX derivatives, **5–8**, are found in the typical ^4^C_1_ conformation (**A**, Fig. 8). This might be an explanation for the exceptional ligand properties as well as the selectivity of C-1-substituted DIX derivatives, which has been observed previously by others and us for similar alkyl-iminoxylitols [30, 34, 36–40].

We are interested in the synthesis of iminosugar-based glycomimetics as tools for profiling and as ligands for modulating GCase activity. Consequently, we want to develop a simple and convenient approach towards N-modified DIX-based building blocks locked in the ^1^C_4_ conformation which carry a substituent suitable for further modifications for different applications taking advantage of the exceptional ligand properties of this system.

## Results and discussion

For this study, we had to take two considerations into account. We wanted to investigate which modification pattern is best for ligand properties, modification at position C-1 or at the ring nitrogen. In addition, we were looking for a suitable functional group at the terminus of the handle which would allow further functionalisation for different applications, including introduction of reporter groups such as fluorescent dyes or click chemistry features. We decided to introduce either an ester group or an imidazole residue. Both functional groups have been found to be suitable for ligand properties of GCase [34, 36].

For the synthesis of the C-1-modified DIX compounds, 1-*C*-ethenyliminoxylitol derivative **16**, which has been synthesized previously by a Staudinger/aza-Wittig/Grignard reaction sequence [40], served as suitable starting material (Scheme 1). Ozonolysis of compound **16** followed by a Horner–Wadsworth–Emmons reaction employing triethyl phosphonoacetate gave 1-*C*-(ethyloxycarbonyl-2-ethenyl)iminoxylitol derivative **17** in 78% over two steps. The double bond was reduced employing Pd/BaSO_4_ as catalyst under hydrogen atmosphere to provide compound **18** in 45% which, after the final deprotection under hydrogenolytical conditions, furnished (*1R*)-1-*C*-ethyloxycarbonylethyl-1,5-dideoxy-1,5-imino-d-xylitol (**19**) in 72%. As expected, this compound exhibits the ^1^C_4_ conformation according to the NMR analysis, coupling constants of protons along the sugar ring exhibit characteristic values in the range of 3–5 Hz as are typical for this conformation.

**Figure:**
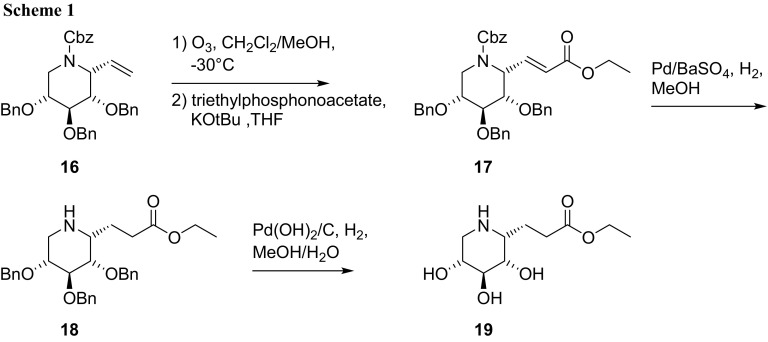

For the introduction of the histamine moiety (Scheme 2), imine **18** was protected with a carboxybenzyl group (Cbz) at the ring nitrogen to give compound **20**. The terminal ester group was saponified employing NaOH to furnish 1-*C*-propionic acid derivative **21**, which was used without purification for the coupling step employing histamine dihydrochloride, (1-cyano-2-ethoxy-2-oxoethylidenaminooxy)dimethylaminomorpholinocarbenium hexafluorophosphate (COMU) and *N*,*N*-diisopropylethylamine (DIEA) as coupling cocktail to give protected (*1R*)-1-*C*-(imidazo-4-yl)ethylaminocarbonylethyliminoxylitol **22** in 75% yield. Final deprotection under hydrogenolytic conditions gave the imidazole-modified iminoxylitol **23** in a yield of 78%. As expected, also this compound features the ^1^C_4_ conformation according to the NMR analysis, coupling constants of protons along the sugar ring exhibit characteristic values in the range of 3–5 Hz.

**Figure:**
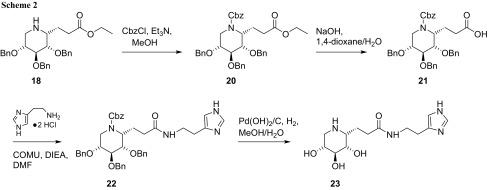

To install the same modification patterns, an ester as well as the imidazole group, at the ring nitrogen, the double bond in protected 1-*C*-ethenyliminoxylitol **16** was reduced employing Pd/BaSO_4_ as catalyst under hydrogen atmosphere (Scheme 3). Under the same reaction conditions, the *N*-Cbz protecting group was cleaved off to give benzyl-protected (*1R*)-1-*C*-ethyliminoxylitol **24** in 81% yield. Introduction of the methoxycarbonylpentyl group at the ring nitrogen was achieved by employing methyl 6-iodohexanoate and sodium carbonate as base in DMF to give N-alkylated iminoxylitol **25** in 67% yield. No formation of a quaternary ammonium ion by double alkylation of the ring nitrogen has been observed during this reaction. Final deprotection of the benzyl groups under hydrogenolytical conditions gave (*1R*)-1-*C*-ethyl-*N*-methoxycarbonylpentyliminoxylitol (**26**) in 88% yield. Also compounds **24**–**26** were found in the ^1^C_4_ conformation exclusively, due to NMR analysis. Likely, the ethyl group at the position C-1 is being responsible for this finding.

**Figure:**
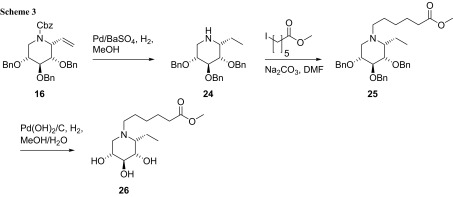

The introduction of the imidazole moiety was conducted accordingly to the synthesis of compound **23** (Scheme 4). Saponification of the methyl ester of compound **25** followed by coupling of the histamine moiety led to protected imidazole-modified iminosugar derivative **27**. Final debenzylation by hydrogenolysis gave (*1R*)-1-*C*-ethyl-*N*-(imidazo-4-yl)ethylethylaminocarbonylpentyliminoxylitol (**28**) in 86% yield. Accordingly, all compounds in this series were also found to adopt the ^1^C_4_ conformation by NMR analysis. The coupling constants of protons along the sugar ring exhibit characteristic values in the range of 3–5 Hz as are typical for this conformation.

**Figure:**
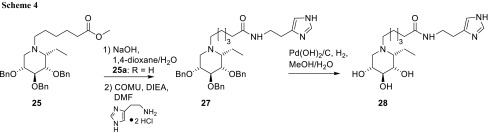

For the biological evaluation of the synthesized DIX derivatives **19**, **23**, **26**, and **28,** we have probed a series of standard glycoside hydrolases, including *β*-glucosidase from *Agrobacterium* sp. (ABG), *β*-galactosidase from *E. coli*, Fabrazyme (commercial recombinant human lysosomal *α*-galactosidase), *α*-glucosidase *S. cerevisiae*, and human *β*-glucocerebrosidase GCase, to investigate ligand activity as well as selectivity. All compounds were found highly selective inhibitors of *β*-glucosidases and showed practically no detectable interaction with *α*-glucosidase (*S. cer.*), *β*-Gal (*E. coli*), as well as human *α*-Gal (Fabrazyme), respectively, confirming the findings of other groups mentioned above. Both imidazole-modified compounds, **23** (*K*_i_ value 1.1 µM) as well as **28** (4.1 µM), showed better inhibitory activity for GCase compared to the ester-modified iminoxylitols **19** (*K*_*i*_ value 5.1 µM) and **26** (57 µM). Concerning our question regarding the modification pattern, we have obtained a very clear picture: 1-*C*-modified iminoxylitols **19** and **23** did not distinguish in their ligand properties between *β*-Glu from ABG and GCase with *K*_i_ values in the same low µM range. In contrast, the ring nitrogen-modified compounds **26** and **28** showed excellent selectivity, with *K*_i_ values of 57 and 4.1 µM, respectively, for GCase. No detectable inhibition of **26** as well as **28** was found with the other enzymes investigated, including *β*-Glu ABG. This increase in selectivity might be explained by the fact that compounds **26** and **28** combine the advantages of both features, the ethyl group at position C-1 locking the structure in the ^1^C_4_ conformation as well as the lipophilic substituents at the ring nitrogen. The former has been implied for favorable ligand properties and the fitting into the active site of GCase. The latter interacts with the lipophilic entrance to the active site of GCase mimicking the ceramide residue of the natural substrate glucosyl ceramide. Compounds **26** and **28** will serve as building blocks for further functionalisation as proposed.

**Table 1:** *K*_i_ values [μM] of compounds with ABG = *β*-glucosidase/*β*-galactosidase from *Agrobacterium* sp.; *β*-galactosidase from *E. coli;* Fabrazyme = commercial recombinant human lysosomal *α*-galactosidase; *α*-glucosidase *S. cerevisiae;* human *β*-glucocerebrosidase Gaucher; N.I. = no inhibition or weak inhibition with estimated *K*_i_ values higher than 1 mM

Enzyme	Compound			
			
*β*-Glu, ABG	2.1	1.6	N.I.	N.I.
*β*-Gal, *E. coli*	N.I.	N.I.	N.I.	N.I.
*α*-Gal, Fabrazyme	N.I.	N.I.	N.I.	N.I.
*α*-Glu, *S. cer.*	N.I.	N.I.	N.I.	N.I.
*β*-Glu, Gaucher	5.1	1.1	57	4.1

## Conclusion

We have investigated which position of substitution at the iminoxylitol scaffold for the introduction of further modifications is favorable, the ring nitrogen or position C-1. Therefore, we have synthesized two compounds in both patterns, one carrying a terminal ester group, compounds **19** and **26**, and the other presenting an imidazole motif, compounds **23** and **28**, for further modification. All four compounds were biologically evaluated with a series of standard glycosidases including human lysosomal *β*-glucocerebrosidase (GCase). Compounds **19** and **23**, with the modification at position C-1 of the DIX scaffold, showed excellent selectivity towards *β*-glucosidases; however, both did not discriminate *β*-Glu from ABG and human lysosomal GCase, with *K*_i_ values found in the low µM range. Compounds **26** and **28**, carrying the modifications at the ring nitrogen and additionally an ethyl group at position C-1, turned out to interact exclusively with human lysosomal GCase with *K*_i_ values of 57 and 4.1 µM, respectively. No detectable inhibition for any other enzyme included in this study has been observed. Thus, DIX-based scaffolds **26** and **28** are excellent building blocks for further modifications customized for different applications, such as for ligands to modulate and tools for profiling GCase activity.

## Experimental

Optical rotations were measured at 20 °C on a Perkin Elmer 341 polarimeter at a wavelength of 589 nm and a path length of 10 cm. NMR spectra were recorded on a Varian INOVA 500 operating at 499.82 MHz (^1^H), and at 125.894 MHz (^13^C) or on a Bruker Ultra-shield spectrometer at 300.36 and 75.53 MHz, respectively. CDCl_3_ was employed for protected compounds and methanol-*d*_4_ or D_2_O for unprotected iminoxylitols. Carbon and hydrogen numbering in NMR spectra was conducted in analogy to carbohydrate nomenclature and clockwise, starting with the pseudo anomeric position carbon as C-1. Chemical shifts are listed in delta employing residual, non-deuterated solvent as the internal standard. Signals were assigned unambiguously by COSY, HSQC, as well as APT analysis. The signals of the protecting groups as well as of the N-substituents were found in the expected regions and are only listed explicitly when overlapping with important spectral features of the respective compound. MALDI-TOF mass spectrometry was performed on a Micromass TofSpec 2E Time-of-Flight Mass Spectrometer. Analytical TLC was performed on precoated aluminum plates silica gel 60 F254 (E. Merck 5554) and detected with UV light (254 nm). For staining, a solution of 9 g vanillin in a mixture of 950 cm^3^ H_2_O/750 cm^3^ EtOH/120 cm^3^ H_2_SO_4_ or ceric ammonium molybdate (100 g ammonium molybdate/8 g ceric sulfate in 1 dm^3^ 10% H_2_SO_4_) was employed followed by heating on a hotplate. For column chromatography, silica gel 60 (230–400 mesh, E. Merck 9385) or silica gel 60 (Acros Organics, AC 24036) were used.

Kinetic studies were performed at 37 °C in an appropriate buffer using a known concentration of enzyme (specific conditions depicted below). *K*_i_ determinations were performed using the corresponding 4-nitrophenyl *α*- or *β*-D-glycopyranoside as substrate. In a typical assay, the enzyme was incubated with different inhibitor concentrations for up to 5 min before initiating the reaction by the addition of substrate. The initial reaction rate was measured by monitoring the increase in absorbance at 400 nm for up to 10 min. *K*_i_ determinations were performed using at least two different substrate concentrations. For each inhibitor, a range of four-to-six inhibitor concentrations bracketing the *K*_i_ value ultimately determined was used for each substrate concentration. Dixon plots (1/*v* vs. [I]) were constructed to validate the use of the competitive inhibition model. The data were then fitted using non-linear regression analysis with Grafit 7.0. Specific assay conditions for each enzyme: *Agrobacterium sp. β*-glucosidase was expressed and purified recombinantly in *E. coli* as previously described [43]: 50 mM sodium phosphate buffer (pH 7) using 1.85 × 10^−4^ mg/cm^3^ of enzyme (*K*_m_ = 4.1 mM) [41, 42]; *E.coli* lac *z β*-galactosidase (Sigma-Aldrich): 50 mM sodium phosphate, 1.0 mM MgCl_2_ (pH 7) using 6.4 × 10^−4^ mg/cm^3^ of enzyme (*K*_m_ = 60 μM); Fabrazyme (acid *α*-galactosidase, generously gifted by Dr Lorne Clarke, Department of Medical Genetics, University of British Columbia): 20 mM sodium citrate, 50 mM sodium phosphate, 1.0 mM tetrasodium EDTA, 0.25% v/v Triton X-100^®^, and 0.25% w/v taurocholic acid buffer (pH 5.5) using 5 × 10^−5^ mg/cm^3^ of enzyme (*K*_m_ = 0.65 mM); *S. cerevisiae α*-glucosidase (Sigma-Aldrich): 50 mM sodium phosphate buffer (pH 7) using 5 × 10^−3^ mg/cm^3^ of enzyme (PNP *α*-Glc, *K*_m_ = 0.75 mM); *β*-Glucocerebrosidase (GCase, generously gifted by Dr. Lorne Clarke, Department of Medical Genetics, University of British Columbia): 20 mM citric acid, 50 mM sodium phosphate, 1 mM tetrasodium EDTA, 0.25% v/v Triton X-100, and 0.25% w/v taurocholic acid (pH 7.0) (*K*_m_ = 1.1 mM).

### (1*R*)-2,3,4-Tri-*O*-benzyl-*N*-(benzyloxycarbonyl)-1-*C*-(ethyloxycarbonylethenyl)-1,5-dideoxy-1,5-imino-d-xylitol (**17**, C_39_H_41_NO_7_)

Compound **16** [40] (550 mg, 0.98 mmol) was dissolved in 100 cm^3^ CH_2_Cl_2_/MeOH (1/1, v/v) and stirred under an atmosphere of ozone at − 30 °C until no starting material was detected on TLC (cyclohexane/EtOAc = 2/1, v/v). N_2_ was bubbled through the reaction mixture to remove ozone traces and 200 mm^3^ dimethylsulfide was added to the reaction mixture, which was stirred for 45 min, followed by concentration under reduced pressure. The resulting colorless oil was added dropwise to a prepared solution of 330 mg KO*t*Bu (2.90 mmol, 3 eq) and 580 mm^3^ triethylphosphonoacetate (2.90 mmol, 3 eq) in 50 cm^3^ THF. Upon consumption of the starting material (detected by TLC: cyclohexane/EtOAc = 2/1, v/v), CH_2_Cl_2_ was added and extracted with 2 N HCl and satd. NaHCO_3_ solution. The organic phase was dried over Na_2_SO_4_ and concentrated under reduced pressure. Purification utilizing silica gel chromatography (cyclohexane/EtOAc = 10/1, v/v) gave compound **17** (500 mg) with a yield of 78% as colorless oil. *R*_*f*_ = 0.55 (cyclohexane/EtOAc = 2/1, v/v). MS: *m/z* calcd. for C_39_H_41_NO_7_Na 658.2781, found 658.2762. Due to two pronounced rotameric populations of the *N*-Cbz group as well as a mixture of E/Z isomers of the double bond, signal splitting as well as signal overlapping in the respective NMR spectra have been observed leading to poor resolution. The respective peaks, however, are observed in the expected region.

### (1*R*)-2,3,4-Tri-*O*-benzyl-1-*C*-(ethyloxycarbonylethyl)-1,5-dideoxy-1,5-imino-d-xylitol (**18**, C_31_H_37_NO_5_)

Compound **17** (1.4 g, 2.2 mmol) was dissolved in 30 cm^3^ MeOH, Pd/BaSO_4_ was added, and the reaction mixture stirred under hydrogen atmosphere until the starting material was not detectable by TLC (cyclohexane/EtOAc = 2/1, v/v). The reaction mixture was filtered and concentrated under reduced pressure. Compound **18** (500 mg) was purified utilizing silica gel chromatography (cyclohexane/EtOAc = 1/1, v/v) and isolated in 45% yield as colorless oil. *R*_*f*_ = 0.10 (cyclohexane/EtOAc = 1/1, v/v); MS: *m/z* calcd. for C_31_H_37_NO_5_Na 526.2569, found 526.2639; ^1^H NMR (300 MHz, CDCl_3_): *δ* = 7.32–7.10 (m, 15H, Ph), 4.53–4.40 (m, 6H, C*H*_2_Ph), 4.05 (q, 2H, H-9), 3.68 (dd, *J*_3,2_ = 5.7 Hz, *J*_3,4_ = 5.5 Hz, 1H, H-3), 3.33 (dd, *J*_2,1_ = 4.5 Hz, 1H, H-2), 3.32 (ddd, *J*_4,5_ = 5.6 Hz, 1H, H-4), 2.96–2.87 (m, 2H, H-1, H-5e), 2.81 (dd, *J*_5a,5e_ = 13.5 Hz, 1H, H-5a), 2.40–2.19 (m, 2H, H-7), 1.85–1.75 (m, 2H, H-6), 1.17 (t, 3H, H-10) ppm; ^13^C NMR (75.5 MHz, CDCl_3_): *δ* = 173.9 (C-8), 138.7, 138.6, 138,5 (3C, 3xC_q_), 128.5–127.7 (Ph), 78.2 (C-4), 77.4 (C-2), 76.5 (C-3), 73.9, 72.4, 72.0 (3C, 3 × *C*H_2_Ph), 60.4 (C-9), 54.5 (C-1), 44.6 (C-5), 31.5 (C-7), 24.0 (C-6), 14.4 (C-10) ppm.

### (1*R*)-1-*C*-(Ethyloxycarbonylethyl)-1,5-dideoxy-1,5-imino-d-xylitol (**19**, C_10_H_19_NO_5_)

Compound **18** (150 mg, 0.30 mmol) was dissolved in MeOH/H_2_O (1/1, v/v) and Pd(OH)_2_ on activated charcoal was added to the solution. The reaction mixture was stirred under hydrogen atmosphere until the starting material was consumed TLC (cyclohexane/EtOAc = 1/2, v/v). The reaction mixture was filtered, concentrated under reduced pressure and the obtained oil was purified utilizing silica gel chromatography (CHCl_3_/MeOH/concd NH_4_OH = 3/1/0.01, v/v/v). Compound **19** (50 mg) was obtained in 72% yield as colorless oil. *R*_*f*_ = 0.80 (CHCl_3_/MeOH/concd NH_4_OH = 1/1/0.25, v/v/v); MS: *m/z* calcd. for C_10_H_19_NO_5_Na 256.1161, found 256.1188; $\left[ a \right]_{D}^{20}$ = − 13.8 (*c* = 1.2, H_2_O); ^1^H NMR (300 MHz, D_2_O): *δ* = 4.10 (q, 2H, H-9), 3.99–3.94 (m, 2H, H-3, H-4), 3.88 (dd, *J*_1,2_ = 3.6 Hz, *J*_2,3_ = 4.6 Hz, 1H, H-2), 3.46 (ddd, *J*_1,2_ = 1.3 Hz, 1H, H-1), 3.36 (dd, *J*_5a,4_ = 2.2 Hz, *J*_5e,5a_ = 13.8 Hz, 1H, H-5a), 3.24 (dd, *J*_5e,4_ = 1.6 Hz, 1H, H-5a), 2.58–2.40 (m, 2H, H-7), 2.08–1.91 (m, 2H, H-6), 1.18 (t, 3H, H-10) ppm; ^13^C NMR (75.5 MHz, D_2_O): *δ* = 174.9 (C-8), 67.5 (C-2), 67.0 (C-3), 66.1 (C-4), 62.1 (C-9), 54.3 (C-1), 45.5 (C-5), 29.4 (C-7), 23.0 (C-6), 13.4 (C-10) ppm.

### (1*R*)-2,3,4-Tri-*O*-benzyl-*N*-(benzyloxycarbonyl)-1-*C*-(eth-yloxycarbonylethyl)-1,5-dideoxy-1,5-imino-d-xylitol (**20**, C_39_H_43_NO_7_)

Compound **18** (750 mg, 1.40 mmol) was dissolved in 20 cm^3^ MeOH and 480 mm^3^ Et_3_N (3.40 mmol, 2.4 eq). CbzCl (250 mm^3^, 1.70 mmol, 1.2 eq) was added and the reaction mixture was stirred at ambient temperature. Upon consumption of the starting material (detected by TLC: cyclohexane/EtOAc = 1/1, v/v), the reaction mixture was concentrated under reduced pressure, dissolved in CH_2_Cl_2_, and extracted with 2 N HCl and sat. NaHCO_3_ solution. The organic layer was dried over Na_2_SO_4_ and concentrated under reduced pressure. Compound **20** (270 mg) was obtained after purification utilizing silica gel chromatography (cyclohexane/EtOAc = 10/1, v/v) in 24% yield as colorless oil. *R*_*f*_ = 0.45 (cyclohexane/EtOAc = 3/1, v/v); ^1^H NMR (300 MHz, CDCl_3_): *δ* = 7.29–7.12 (m, 20H, Ph), 5.03–4.94 (m, 2H, C*H*_2_Cbz), 4.80–4.74 (m, 2H, C*H*_2_Ph), 4.65–4.50 (m, 5H, 2xC*H*_2_Ph, H-1), 4.36–4.26 (m, 1H, H-1, H-5e), 4.09–3.90 (m, 2H, H-9, H-5e), 3.58 (dd, *J*_3,2_ = 9.0 Hz, *J*_3,4_ = 9.2 Hz, 1H, H-3), 3.42 (dd, *J*_2,1_ = 6.1 Hz, 1H, H-2), 3.32 (ddd, *J*_4,5_ = 5.5 Hz, 1H, H-4), 2.65 (dd, *J*_5a,5e_ = 13.1 Hz, 1H, H-5a), 2.22–2.07 (m, 2H, H-7), 1.93–1.72 (m, 2H, H-6), 1.12 (t, 3H, H-10) ppm; ^13^C NMR (75.5 MHz, CDCl_3_): *δ* = 173.1 (d, C-8), 155.6 (d, Cbz), 138.9, 138.2, 136.4 (C_q_-Ph), 128.7–127.0 (Ph), 82.0 (d, C-3), 79.6 (d, C-2), 78.2 (C-4), 75.8, 73.2, 72.8 (d, *C*H_2_Ph), 67.7 (d, *C*H_2_Cbz), 60.5 (d, C-9), 52.7 (d, C-1), 40.9 (d, C-5), 30.7 (C-7), 19.9 (d, C-6), 14.3 (C-10) ppm. Due to two pronounced rotameric populations (**20**) of the *N*-Cbz group, signal splitting in the respective NMR spectra has been observed leading to somehow poor resolution of NMR spectra.

### (1*R*)-2,3,4-Tri-*O*-benzyl-*N*-(benzyloxycarbonyl)-1-*C*-(carboxy-ethyl)-1,5-dideoxy-1,5-imino-d-xylitol (**21**, C_37_H_39_NO_7_)

Compound **20** (220 mg, 0.35 mmol) was dissolved in 20 cm^3^ dioxane/H_2_O (1/1, v/v) and 1 cm^3^ of a 3 M NaOH solution was added dropwise. After consumption of the starting material (detected by TLC: cyclohexane/EtOAc = 3/1, v/v), the reaction mixture was acidified with 2 N HCl and extracted with EtOAc. The combined organic layers were dried over Na_2_SO_4_ and concentrated under reduced pressure. Compound **21** (230 mg) was obtained as slightly yellow oil containing minor amounts of impurities and has been employed for the next step without further purification. *R*_*f*_ = 0.60 (EtOAc); ^1^H NMR (300 MHz, MeOH-*d*_*4*_): *δ* = 7.27–7.08 (m, 20H, Ph), 5.02–4.90 (m, 2H, C*H*_2_Cbz), 4.81–4.72 (m, 2H, C*H*_2_Ph), 4.57–4.35 (m, 5H, 2xC*H*_2_Ph, H-1), 4.19 (dd, *J*_5e,4_ = 5.2 Hz, *J*_5e,5a_ = 13.3 Hz, 1H, H-5e), 4.03 (dd, 1H, H-5a), 3.51 (dd, *J*_3,2_ = 8.5 Hz, *J*_3,4_ = 9.1 Hz, 1H, H-3), 3.35–3.18 (m, 2H, H-2, H-4), 2.18–1.63 (m, 4H, H-6, H-7) ppm; ^13^C NMR (75.5 MHz, CDCl_3_): *δ* = 179.1 (d, C-8), 155.7 (d, Cbz), 138.9, 138.1, 136.5 (C_q_-Ph), 128.7–127.7 (Ph), 82.1 (d, C-3), 79.9 (d, C-2), 78.3 (C-4), 75.8, 73.3, 72.9 (*C*H_2_-Ph), 67.7 (d, *C*H_2_-Cbz), 52.8 (d, C-1), 40.8 (d, C-5), 30.7 (C-7), 19.7 (d, C-6) ppm. Due to two pronounced rotameric populations (**21**) of the *N*-Cbz group, signal splitting in the respective NMR spectra has been observed.

### (1*R*)-2,3,4-Tri-*O*-benzyl-*N*-(benzyloxycarbonyl)-1-*C*-[(imidazo-4-yl)ethylaminocarbonylethyl]-1,5-dideoxy-1,5-imino-d-xylitol (**22**, C_42_H_46_N_4_O_6_)

Compound **21** (340 mg, 0.57 mmol) was dissolved in 20 cm^3^ DMF. COMU (490 mg, 1.14 mmol, 2 eq) and 400 mm^3^ DIEA (2.33 mmol, 4 eq) were added, and the reaction mixture was stirred for 30 min at ambient temperature. Histamine dihydrochloride (160 mg, 0.86 mmol, 1.5 eq) was added and the reaction mixture was stirred until the starting material was consumed, TLC (EtOAc/MeOH = 10/1, v/v). The reaction mixture was concentrated under reduced pressure and purified by silica gel chromatography (EtOAc/MeOH = 10/1, v/v) to give compound **22** (300 mg) in 75% yield. *R*_*f*_ = 0.60 (CHCl_3_/MeOH/concd. NH_4_OH = 6/1/0.01, v/v/v); MS: *m/z* calcd. for C_42_H_46_N_2_O_6_Na 725.3315, found 725.3347. Due to two pronounced rotameric populations (**22**) of the *N*-Cbz group, signal splitting in the respective NMR spectra has been observed leading to poor resolution of the NMR spectra.

### (1*R*)-1-*C*-[(Imidazo-4-yl)ethylaminocarbonylethyl]-1,5-dideoxy-1,5-imino-d-xylitol (**23**, C_13_H_22_N_4_O_4_)

Compound **22** (300 mg, 0.43 mmol) was dissolved in 15 cm^3^ MeOH/H_2_O (1/1, v/v), Pd(OH)_2_ on activated charcoal was added and the reaction mixture was stirred under hydrogen atmosphere. Upon consumption of the starting material (detected by TLC: CHCl_3_/MeOH/concd NH_4_OH = 6/1/0.01, v/v/v), the reaction mixture was filtered and concentrated under reduced pressure. After purification by silica gel chromatography (CHCl_3_/MeOH/concd NH_4_OH = 3/1/0.25, v/v/v) compound **23** (100 mg) was obtained as colorless oil in 78% yield. *R*_*f*_ = 0.50 (CHCl_3_/MeOH/concd NH_4_OH = 1/1/0.25, v/v/v); MS: *m/z* calcd. for C_13_H_22_N_4_O_4_Na 321.1539, found 321.1567; $\left[ a \right]_{D}^{20}$ = − 6.5 (*c* = 1, H_2_O); ^1^H NMR (300 MHz, D_2_O): *δ* = 7.83 (s, 1H, H-13), 6.92 (s, 1H, H-12), 3.77 (ddd, *J*_3,4_ = 4.8 Hz, *J*_3,2_ = 5.2 Hz, 1H, H-3), 3.75 (dd, *J*_4,5e_ = 3.8 Hz, *J*_4,5a_ = 4.6 Hz, 1H, H-4), 3.69 (dd, *J*_1,2_ = 3.2 Hz, 1H, H-2), 3.36 (t, 2H, H-9), 3.13 (ddd, 1H, H-1), 3.21 (dd, 1H, H-5e), 2.97 (dd, *J*_5e,5a_ = 13.6 Hz, 1H, H-5a), 2.72 (t, 2H, H-10), 2.22 (t, 2H, H-7), 1.89–1.70 (m, 2H, H-6) ppm; ^13^C NMR (75.5 MHz, D_2_O): *δ* = 175.1 (C-8), 135.1 (C-13), 133.6 (C-11), 116.8 (C-12), 68.9 (2C, C-2, C-3), 67.0 (C-4), 54.6 (C-1), 44.8 (C-5), 38.7 (C-9), 31.7 (C-7), 25.4 (C-10), 23.3 (C-6) ppm.

### (1*R*)-2,3,4-Tri-*O*-benzyl-1-*C*-ethyl-1,5-dideoxy-1,5-imino-d-xylitol (**24**, C_28_H_33_NO_3_)

Compound **16** [40] (1.2 g, 2.13 mmol) was dissolved in 20 cm^3^ MeOH. Pd/BaSO_4_ was added and the reaction mixture was stirred under hydrogen atmosphere. Upon consumption of the starting material (detected by TLC: cyclohexane/EtOAc = 3/1, v/v) the reaction mixture was filtered and concentrated under reduced pressure. Compound **24** (740 mg) was obtained with a yield of 81% as colorless oil. *R*_*f*_ = 0.2 (cyclohexane/EtOAc = 3/1, v/v); $\left[ a \right]_{D}^{20}$ = − 1.1 (*c* = 1.0, CHCl_3_); ^1^H NMR (300 MHz, CDCl_3_): *δ* = 7.35–7.07 (m, 15H, Ph), 4.65–4.35 (m, 6H, C*H*_*2*_Ph), 3.67 (dd, *J*_3,4=3,2_ = 5.7 Hz, 1H, H-3), 3.38–3.23 (m, 2H, H-2, H-4), 2.91 (dd, *J*_5e,4_ = 4.1 Hz, *J*_5e,5a_ = 13.4 Hz, 1H, H-5e), 2.89 (dd, *J*_5a,4_ = 5.5 Hz, 1H, H-5a), 2.83–2.76 (m, *J*_1,2_ = 3.9 Hz, 1H, H-1), 1.55–1.42 (m, 2H, H-6), 0.81 (t, 3H, H-7) ppm; ^13^C NMR (75.5 MHz, CDCl_3_): *δ* = 138.7 (3x C_q_), 128.4–127.6 (Ph), 78.1 (C-2), 76.9 (C-3), 76.6 (C-4), 73.8, 72.2, 71.9 (3C, 3x *C*H_2_-Ph), 56.5 (C-1), 44.4 (C-5), 21.2 (C-6), 10.8 (C-7) ppm.

### (1*R*)-2,3,4-Tri-*O*-benzyl-1-*C*-ethyl-*N*-(methyloxycarbonylpentyl)-1,5-dideoxy-1,5-imino-d-xylitol (**25**, C_35_H_45_NO_5_)

Compound **24** (740 mg, 1.72 mmol) was dissolved in 20 cm^3^ DMF. 6-Iodohexylmethylester (660 mg, 2.60 mmol, 1.5 eq) and 545 mg Na_2_CO_3_ (5.15 mmol, 3 eq) were added and the reaction mixture was stirred at 60 °C. Upon consumption of the starting material (detected by TLC: cyclohexane/EtOAc = 2/1, v/v), the reaction mixture was concentrated under reduced pressure, dissolved in CH_2_Cl_2_, and extracted with 2 N HCl and satd. NaHCO_3_ solution. The organic layer was dried over Na_2_SO_4_ and concentrated under reduced pressure. Purification by silica gel chromatography gave compound **25** (640 mg) in a yield of 67% as colorless oil. *R*_*f*_ = 0.55 (cyclohexane/EtOAc = 2/1, v/v); $\left[ a \right]_{D}^{20}$ = + 12.7 (*c* = 1.1, CHCl_3_); MS: *m/z* calcd. for C_35_H_45_NO_5_Na 582.3195, found 582.3217; ^1^H NMR (300 MHz, CDCl_3_): *δ* = 7.29–7.16 (m, 15H, Ph), 4.80, 4.74 (2xd, 2H, C*H*_*2*_Ph), 4.66–4.53 (m, 4H, C*H*_2_Ph), 3.59 (s, 3H, H-14), 3.56–3.43 (m, 3H, H-2, H-3, H-4), 2.78–2.61 (m, *J*_1,2_ = 3.6 Hz, *J*_5e,4_ = 4.4 Hz, *J*_5e,5a_ = 12.9 Hz, 2H, H-1, H-5e), 2.52–2.34 (m, *J*_5a,4_ = 5.5 Hz, 3H, H-5a, H-8), 2.22 (t, 2H, H-12), 1.63–1.35 (m, 4H, H-6, H-11), 1.29–1.16 (m, 4H, H-9, H-10), 0.87 (t, 3H, H-7) ppm; ^13^C NMR (75.5 MHz, CDCl_3_): *δ* = 174.3 (C-13), 139.2, 138.8, 138.7 (3C, 3x C_q_), 128.4–127.4 (Ph), 83.2 (C-4), 80.6 (C-2), 78.3 (C-3), 75.5, 73.1, 72.8 (3x *C*H_2_Ph), 61.6 (C-1), 54.2 (C-8), 51.5 (C-14), 48.5 (C-5), 34.2 (C-12), 28.4 (C-9), 26.7 (C-10), 24.9 (C-11), 16.5 (C-6), 13.5 (C-7) ppm.

### (1*R*)-1-*C*-Ethyl-*N*-(methyloxycarbonylpentyl)-1,5-dideoxy-1,5-imino-d-xylitol (**26**, C_14_H_27_NO_5_)

Compound **25** (350 mg, 0.6 mmol, 1 eq) was dissolved in 10 cm^3^ MeOH/H_2_O (1/1, v/v), Pd(OH)_2_/C was added and the reaction mixture was stirred under hydrogen atmosphere at ambient pressure. Upon consumption of the starting material (detected by TLC, eluent: CHCl_3_/MeOH/concd. NH_4_OH = 3/1/0.01, v/v/v), the reaction mixture was filtered, concentrated under reduced pressure, and purified by silica gel chromatography (CHCl_3_/MeOH/concd. NH_4_OH = 10/1/0.01, v/v/v) which gave compound **26** (160 mg) in a yield of 88% as colorless oil. *R*_*f*_ = 0.66 (CHCl_3_/MeOH/concd. NH_4_OH = 3/1/0.01, v/v/v); MS: *m/z* calcd. for C_14_H_27_NO_5_Na 312.1787, found 312.1845; $\left[ a \right]_{D}^{20}$ = + 7.4 (*c* = 1.0, MeOH); ^1^H NMR (300 MHz, MeOH-*d*_*4*_): *δ* = 4.01–4.00 (m, 2H, H-3, H-4), 3.96–3.94 (dd,*J*_1,2_ = 3.6 Hz, *J*_2,3_ = 4.0 Hz, 1H, H-2), 3.69 (s, 3H, H-14), 3.51 (bdd, *J*_5e,4_ = 1.2 Hz, *J*_5e,5a_ = 12.8 Hz, 1H, H-5e), 3.41–3.32 (m, *J*_5a,4_ = 3.3 Hz, 2H, H-1, H-5a), 3.23 (q, 2H, H-8), 2.41 (t, 2H, H-12), 2.00–1.89 (m, 2H, H-6), 1.82–1.67 (m, 4H, H-9, H-11), 1.50–1.40 (m, 2H, H-10), 1.06 (t, 3H, H-7) ppm; ^13^C NMR (75.5 MHz, MeOH-*d*_*4*_): *δ* = 175.7 (C-13), 69.4 (C-4), 69.2 (C-3, C-2), 63.7 (C-1), 54.3 (C-5), 53.7 (C-8), 52.1 (C-14), 34.5 (C-12), 27.2 (C-10), 25.5 (C-11), 23.4 (C-9), 19.8 (C-6), 10.3 (C-7) ppm.

### (1*R*)-2,3,4-Tri-*O*-benzyl-*N*-(carboxypentyl)-1-*C*-ethyl-1,5-dideoxy-1,5-imino-d-xylitol (**25a**, C_34_H_43_NO_5_)

Compound **25** (60 mg, 0.11 mmol) was dissolved in 5 cm^3^ dioxane/H_2_O (1/1, v/v). NaOH solution (3 M, 10 drops) was added and the reaction mixture was stirred until the starting material was consumed (TLC cyclohexane/EtOAc = 2/1, v/v). EtOAc was added and the reaction mixture was washed with 2 N HCl and satd. NaHCO_3_ solution. The organic layer was dried over Na_2_SO_4_ and concentrated under reduced pressure. Compound **25a** (40 mg) was obtained as colorless oil and was used without purification for the next step. *R*_*f*_ = 0.45 (cyclohexane/EtOAc = 1/1, v/v); ^1^H NMR (300 MHz, CDCl_3_): *δ* = 7.35–7.06 (m, 15H, Ph), 4.83–4.43 (m, 6H, C*H*_*2*_Ph), 3.71–3.50 (m, 3H, H-2, H-3, H-4), 3.05–3.34 (m, 2H, H-1, H-5e), 2.68–2.42 (m, 3H, H-5a, H-8), 2.20 (t, 2H, H-12), 1.70–1.11 (m, 8H, H-6, H-9, H-10, H-11), 0.89 (t, 3H, H-7) ppm; ^13^C NMR (75.5 MHz, CDCl_3_): *δ* = 178.2 (C=O), 139.1, 138.7, 138.6 (3x C_q_), 128.6–127.7 (Ph), 81.0, 79.3, 77.3 (C-2, C-3, C-4), 75.2, 73.2, 73,0 (3x *C*H_2_-Ph), 61.0 (C-1), 53.4 (C-8), 48.3 (C-5), 34.6 (C-12), 27.0, 26.6, 24.9 (C-9, C-10, C-11), 16.4 (C-6), 13.4 (C-7) ppm.

### (1*R*)-2,3,4-Tri-*O*-benzyl-1-*C*-ethyl-*N*-[(imidazo-4-yl)ethyl-aminocarbonylpentyl]-1,5-dideoxy-1,5-imino-d-xylitol (**27**, C_39_H_50_N_4_O_4_)

Compound **25a** (400 mg, 0.73 mmol) was dissolved in 20 cm^3^ DMF. COMU (704 mg, 1.46 mmol, 2 eq) and 509 mm^3^ DIEA (2.92 mmol, 4 eq) were added and the reaction mixture was stirred for 30 min. Histamine dihydrochloride (203 mg, 1.10 mmol, 1.5 eq) was added to the reaction mixture and stirred until the starting material was consumed (TLC EtOAc/MeOH = 10/1, v/v). The reaction mixture was concentrated under reduced pressure and purified by silica gel chromatography (CHCl_3_/MeOH/concd. NH_4_OH = 12/1/0.01, v/v/v) to give compound **27** (140 mg) as yellow solid with small impurities. *R*_*f*_ = 0.45 (cyclohexane/EtOAc = 1/1, v/v); MS: *m/z* calcd. for C_39_H_50_N_4_O_4_Na 661.3730, found 661.3705; ^1^H NMR (300 MHz, MeOH-*d*_*4*_): *δ* = 7.67 (s, 1H, H-18), 7.40–7.26 (m, 15H, Ph), 6.90 (s, 1H, H-17), 4.82–4.52 (m, 6H, C*H*_*2*_Ph), 3.82–3.65 (m, 3H, H-2, H-3, H-4), 3.45 (t, 2H, H-14), 3.12–3.00 (m, 2H, H-1, H-5e), 2.92–2.73 (m, 4H, H-5a, H-12, H-15), 2.19 (t, 2H, H-8), 1.79–1.23 (m, 8H, H-6, H-9, H-10, H-11), 0.93 (t, 3H, H-7) ppm; ^13^C NMR (75.5 MHz, MeOH-*d*_*4*_): *δ* = 176.1 (C-13), 139.9, 139.7, 139.6 (3x C_q_), 136.0 (C-18), 135.6 (C-16), 129.5–128.9 (Ph), 118.2 (C-17), 78.5 (C-3), 77.1 (2C, C-2, C-4), 75.5, 73.7, 73.6 (3x *C*H_2_-Ph), 63.0 (C-1), 54.6 (C-12), 50.5 (C-5), 40.3 (C-14), 37.0 (C-8), 27.7, 27.5, 27.0, 26.7 (C-9, C-10, C-11, C-15), 18.1 (C-6), 13.2 (C-7) ppm.

### (1*R*)-1-*C*-Ethyl-*N*-[(imidazo-4-yl)ethylaminocarbonylpentyl]-1,5-dideoxy-1,5-imino-d-xylitol (**28**, C_18_H_32_N_4_O_4_)

Compound **27** (140 mg, 0.22 mmol) was dissolved in 5 cm^3^ MeOH/H_2_O (1/1, v/v), Pd(OH)_2_/C was added and the reaction mixture was stirred under hydrogen atmosphere at ambient pressure. Upon consumption of the starting material (detected by TLC, eluent: CHCl_3_/MeOH/concd. NH_4_OH = 2/1/0.25, v/v/v), the reaction mixture was filtered, concentrated under reduced pressure, and purified by silica gel chromatography (CHCl_3_/MeOH/concd. NH_4_OH = 3/1/0.25, v/v/v), which gave compound **28** (70 mg) in a yield of 86% as colorless oil. *R*_*f*_ = 0.60 (CHCl_3_/MeOH/concd NH_4_OH = 2/1/0.25, v/v/v); MS: *m/z* calcd. for C_18_H_32_N_4_O_4_Na 391.2321, found 391.2384; $\left[ a \right]_{D}^{20}$ = + 5.8 (*c* = 1.04, H_2_O); ^1^H NMR (300 MHz, D_2_O): *δ* = 7.95 (s, 1H, H-18), 6.96 (s, 1H, H-17), 3.87 (ddd, *J*_4.5e_ = 3.1 Hz, *J*_4,5a_ = 6.3 Hz, *J*_4,3_ = 5.6 Hz, 1H, H-4), 3.82 (dd, *J*_2,1_ = 3.5 Hz, *J*_2,3_ = 5.8 Hz, 1H, H-2), 3.75 (dd, 1H, H-3), 3.38 (t, 2H, H-14), 3.27–3.16 (m, 2H, H-1, H-5e), 2.98 (dd, *J*_5a,5e_ = 12.5 Hz, *J*_4,5a_ = 6.4 Hz, 1H, H-5a), 2.93 (t, 2H, H-12), 2.76 (t, 2H, H-15), 2.13 (t, 2H, H-8), 1.79–1.42 (m, 6H, H-6, H-9, H-11), 1.29–1.10 (m, 2H, H-10), 0.92 (t, 3H, H-7) ppm; ^13^C NMR (75.5 MHz, D_2_O): *δ* = 176.6 (C-13), 134.7 (C-18), 133.0 (C-16), 116.8 (C-17), 69.4 (C-3), 68.6 (C-4), 67.6 (C-2), 62.4 (C-1), 52.7 (C-12), 51.8 (C-5), 38.4 (C-14), 35.4 (C-8), 25.4 (C-10), 25.2 (C-9), 24.9 (C-15), 22.9 (C-11), 17.1 (C-6), 10.8 (C-7) ppm.

## Electronic supplementary material

Below is the link to the electronic supplementary material.

Supplementary material 1 (DOCX 948 kb)
