# Supplementary material for: Synthesis of modified 1,5-imino-d-xylitols as ligands for lysosomal β-glucocerebrosidase
Source: Monatsh Chem. 2019 May 11;150(5):831–42. doi: 10.1007/s00706-019-02427-1 (PMC6534063; doi:10.1007/s00706-019-02427-1)

Correspondence to

Tanja M. Wrodnigg

Glycogroup, Institut für Organische Chemie

Technische Universität Graz

Stremayrgasse 9

A-8010 Graz

Austria

email:t.wrodnigg@tugraz.at

**SUPPORTING INFORMATION FOR**

Synthesis of modified 1,5-imino-d-xylitols as ligands for lysosomal β-glucocerebrosidase

**Manuel Zoidl^1^ ● Andreas Wolfsgruber^1^ ● Michael Schalli^1^ ● Seyed A. Nasseri ^2^ ● Patrick Weber^1^● Arnold E. Stütz^1^● Stephen G. Withers^2^ ● Tanja M. Wrodnigg^1^**

^1^Glycogroup, Institute of Organic Chemistry, Graz University of Technology,Stremayrgasse 9, A-8010 Graz, Austria

^2^Chemistry Department, University of British Columbia,2036 Main Mall, Vancouver, BC, Canada V6T 1Z1

***(1R)-2,3,4-Tri-O-benzyl-1-C-ethyloxycarbonylethyl-1,5-dideoxy-1,5-imino-d-xylitol (18).***

^1^H NMR (300 MHz, CDCl_3_):


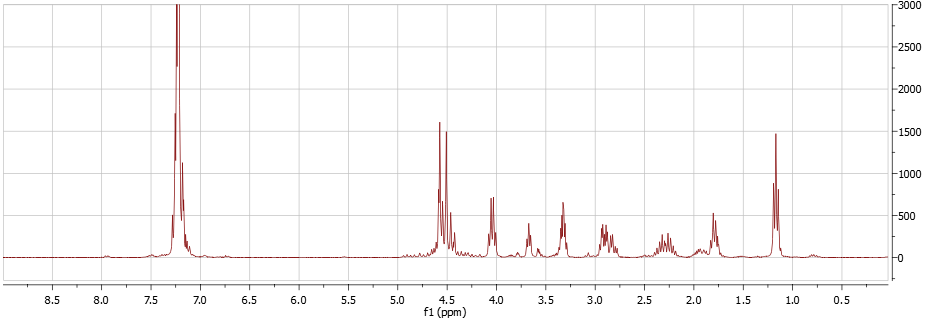


^13^C NMR (75.5 MHz, CDCl_3_):


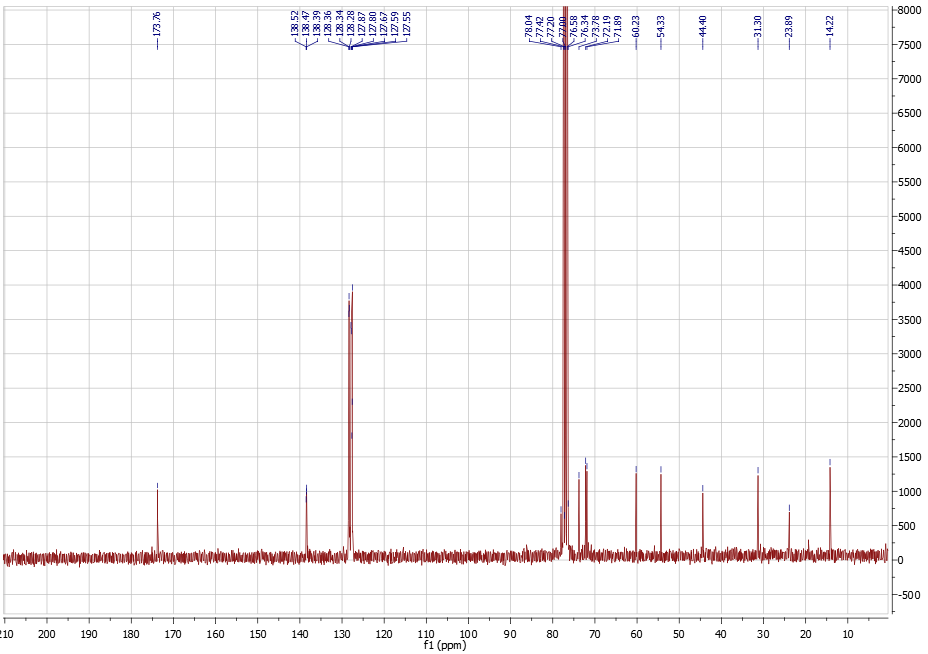

***(1R)-1-C-Ethyloxycarbonylethyl-1,5-dideoxy-1,5-imino-d-xylitol (19).***

^1^H NMR (300 MHz, D_2_O):


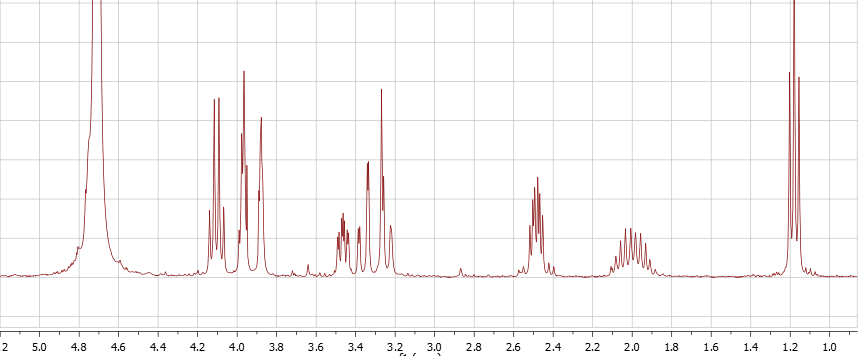


^13^C NMR (75.5 MHz, D_2_O):


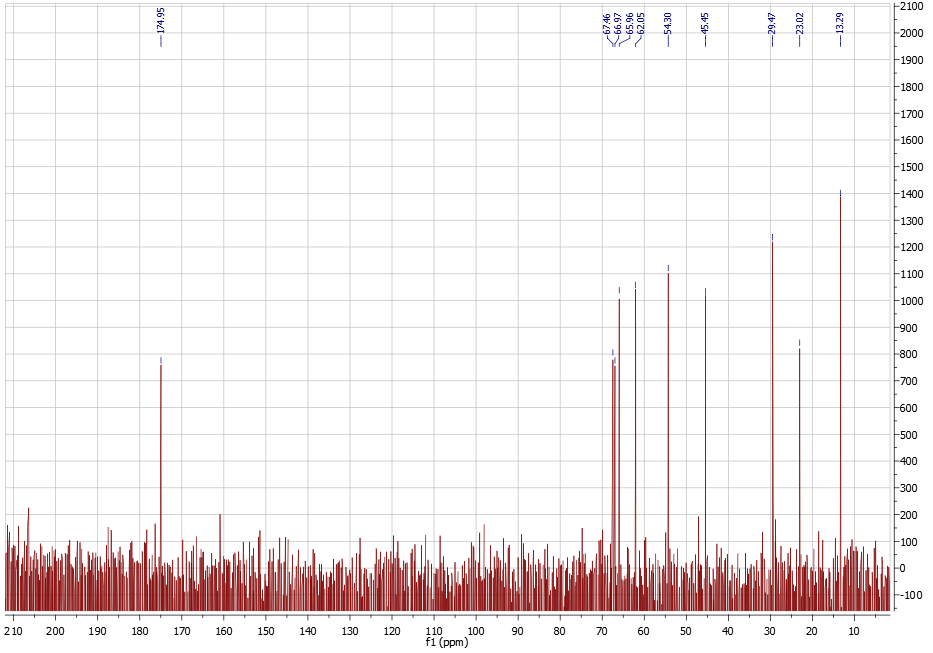

***(1R)-1-C-(Imidazo-4-yl)ethylaminocarbonylethyl-1,5-dideoxy-1,5-imino-d-xylitol (23).***

^1^H NMR (300 MHz, D_2_O):


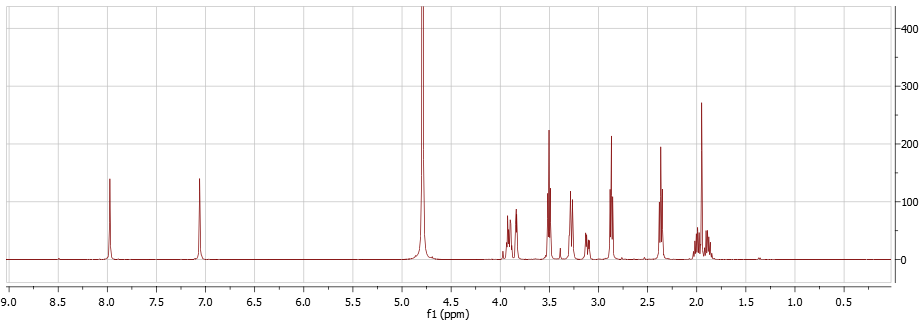


^13^C NMR (75.5 MHz, D_2_O):


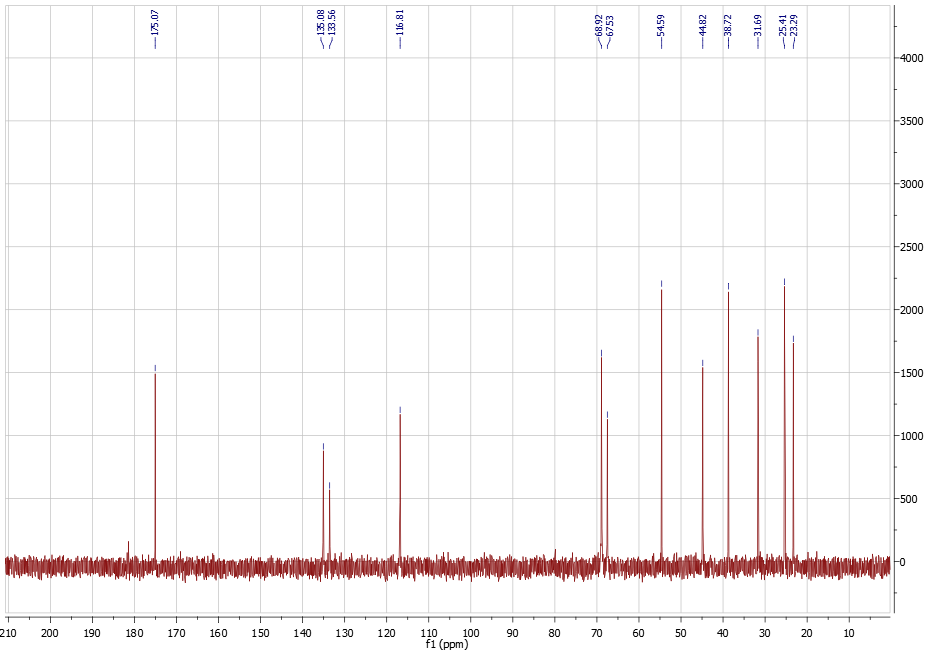

***(1R)-2,3,4-Tri-O-benzyl-1-C-ethyl-1,5-dideoxy-1,5-imino-d-xylitol (24).***

^1^H NMR (300 MHz, CDCl_3_):


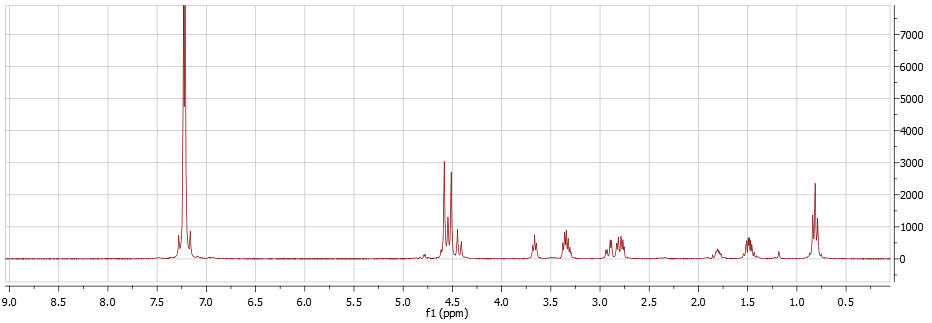


^13^C NMR (75.5 MHz, CDCl_3_):


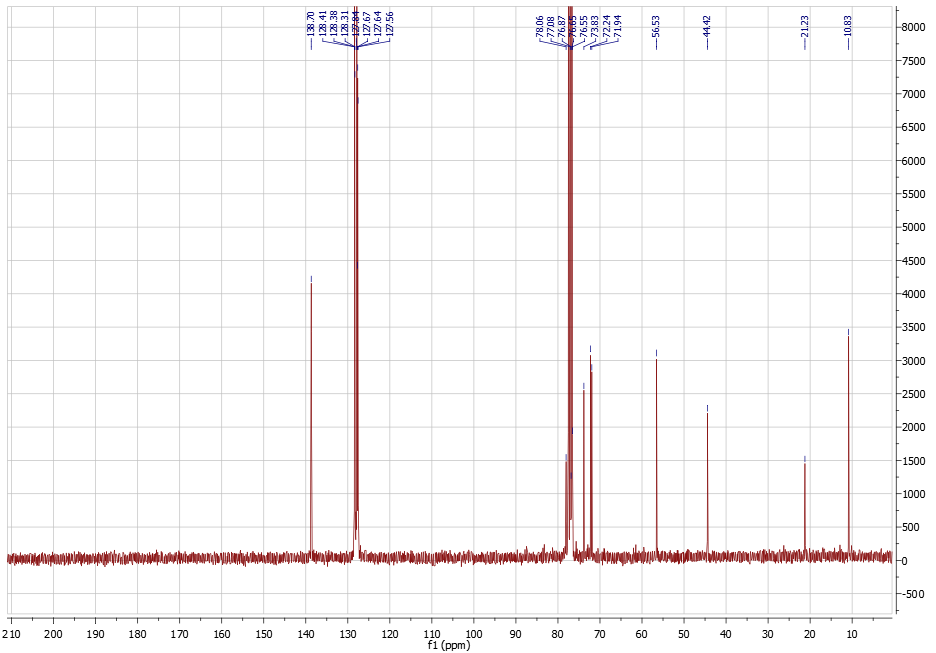

***(1R)-2,3,4-Tri-O-benzyl-1-C-ethyl-N-methyloxycarbonylpentyl-1,5-dideoxy-1,5-imino-d-xylitol (25).***

^1^H NMR (300 MHz, CDCl_3_):


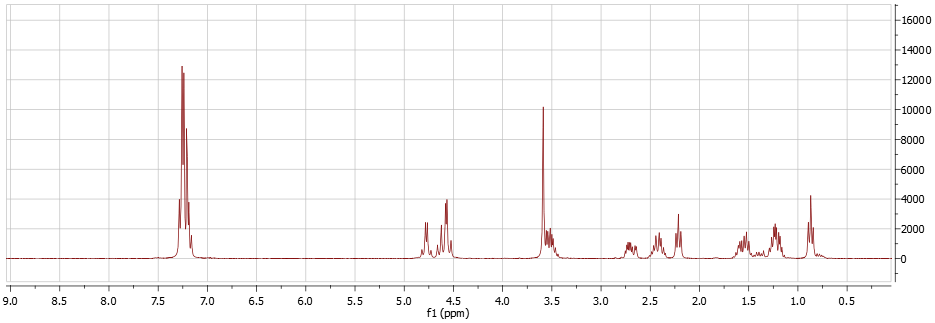


^13^C NMR (75.5 MHz, CDCl_3_):


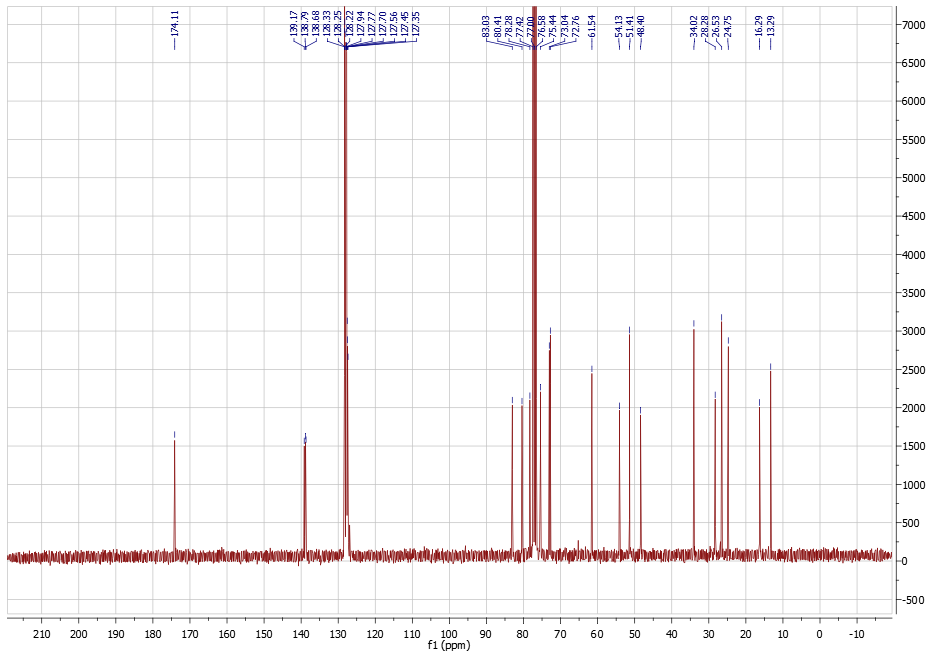

***(1R)-1-C-Ethyl-N-methyloxycarbonylpentyl-1,5-dideoxy-1,5-imino-d-xylitol (26).***

^1^H NMR (300 MHz, MeOH-*d_4_*):


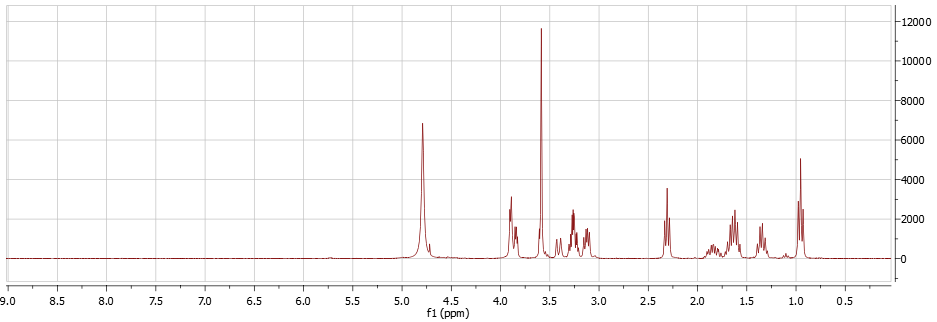


^13^C NMR (75.5 MHz, MeOH-*d_4_*):


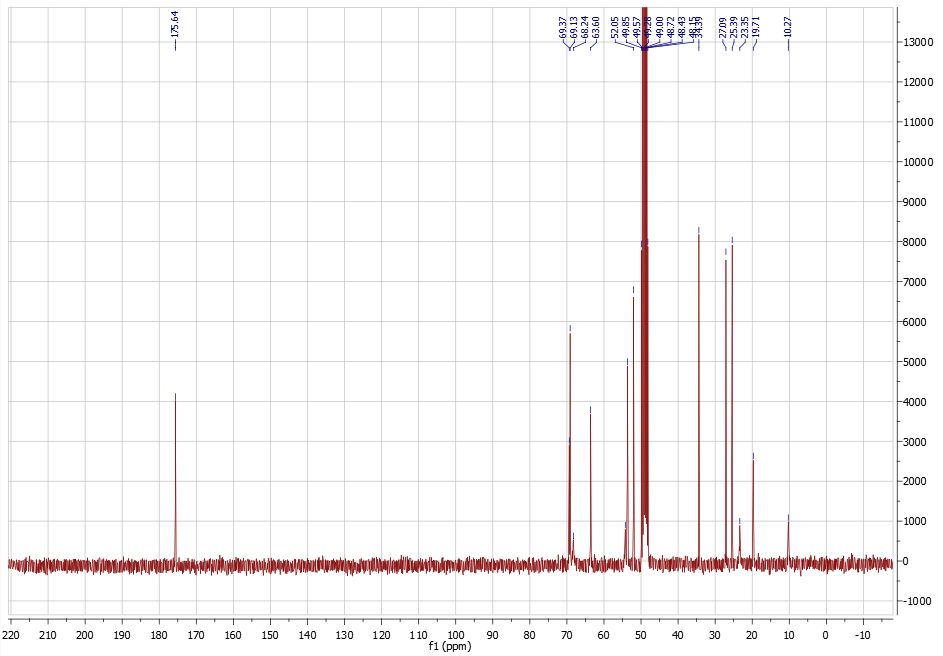

***(1R)-2,3,4-Tri-O-benzyl-N-carboxypentyl-1-C-ethyl-1,5-dideoxy-1,5-imino-d-xylitol (25a).***

^1^H NMR (300 MHz, CDCl_3_):


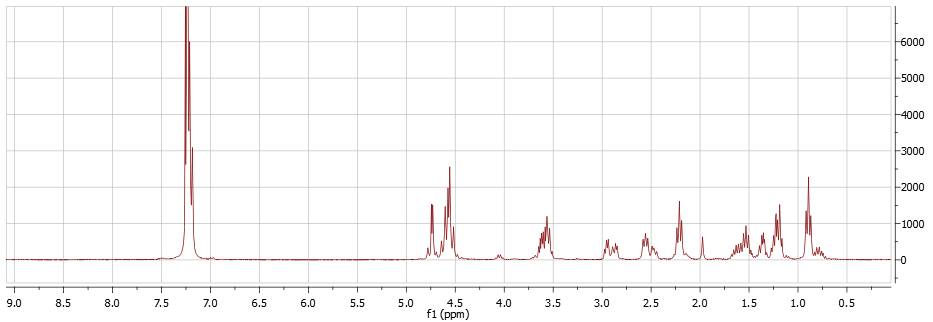


^13^C NMR (75.5 MHz, CDCl_3_):


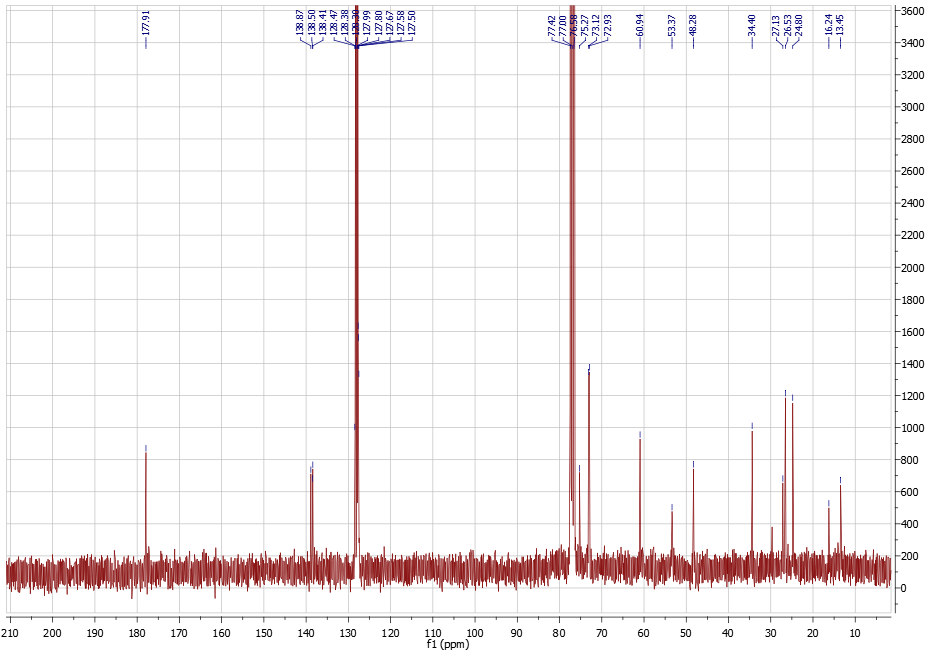

***(1R)-1-C-Ethyl-N-(imidazo-4-yl)ethylaminocarbonylpentyl-1,5-dideoxy-1,5-imino-d-xylitol (28).***

^1^H NMR (300 MHz, D_2_O):


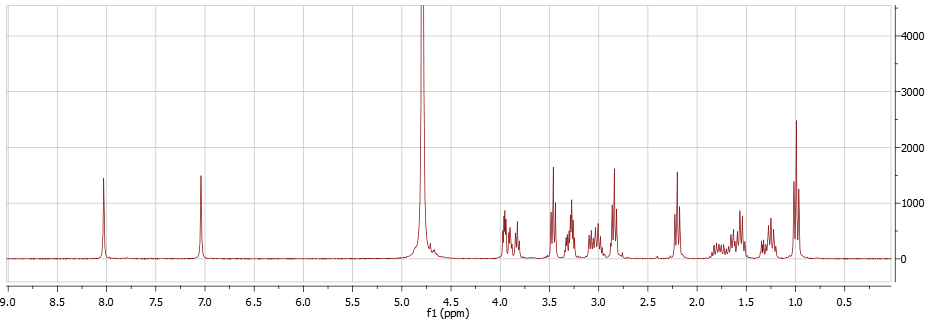


^13^C NMR (75.5 MHz, D_2_O):


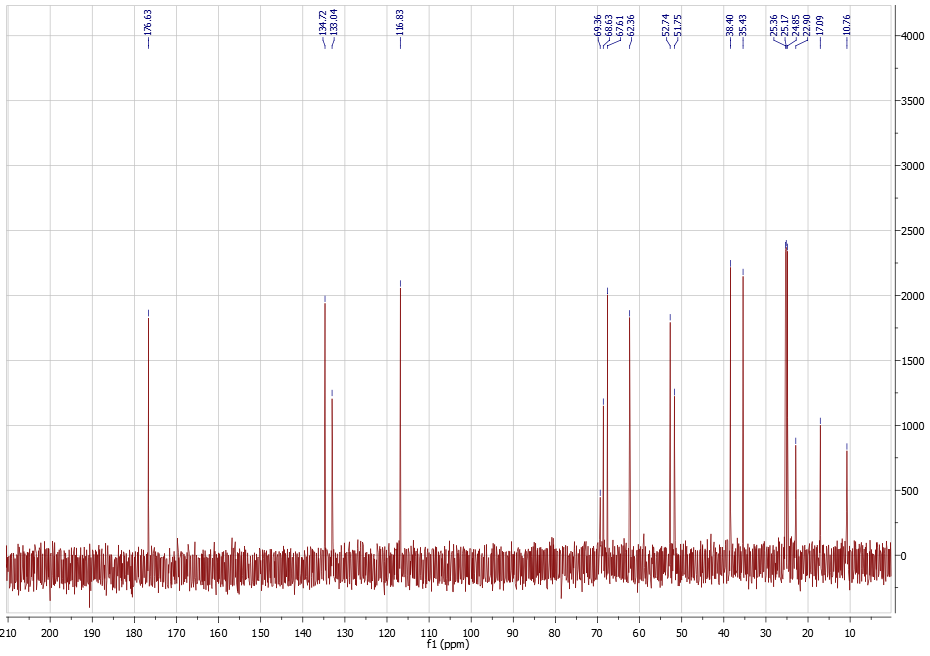

Supplement: Supplementary file 1 — Supplementary material 1 (DOCX 948 kb) [file 706_2019_2427_MOESM1_ESM.docx]
